# Prevalence and timing of *TP53* mutations in del(17p) myeloma and effect on survival

**DOI:** 10.1038/bcj.2017.76

**Published:** 2017-09-15

**Authors:** M Chin, J I Sive, C Allen, C Roddie, S J Chavda, D Smith, P Blombery, K Jones, G L Ryland, R Popat, A Rismani, S D'Sa, N Rabin, R E Gale, K L Yong

**Affiliations:** 1 Department of Haematology, UCL Cancer Institute, London, UK; 2 Department of Haemato-Oncology, St Bartholomew's Hospital, London, UK; 3 Department of Pathology, Peter MacCallum Cancer Centre, Melbourne, Victoria, Australia; 4 Department of Haematology, UCLH, London, UK

The recent remarkable advances in multiple myeloma (MM) therapy and outcomes have had mixed impact on patients with adverse risk genetics, many of whom continue to have inferior outcomes. This applies particularly to deletion of chromosome 17p13 [del(17p)], found in ≈10% of newly diagnosed MM (ND) and at higher prevalence in advanced disease.^1, 2, 3, 4, 5^ The *TP53* gene is located within the minimally deleted region on 17p13, and is thought to confer the adverse risk in a haploinsufficient manner.^6^ The incidence of *TP53* mutations in ND is ≈3% but also increases with disease progression,^7, 8^ and is associated with shorter survival.^9^ Although *TP53* mutations are uncommon in the absence of del(17p), approximately one third of del(17p) MM patients are reported to have a *TP53* mutation, and this increases in refractory disease to more than 50%.^8, 10^

The prognostic impact of *TP53* mutations in del(17p) MM remains unresolved.^2, 10^ We therefore carried out mutational analysis of a series of MM patients who had fluorescence *in situ* hybridization (FISH)-identified del(17p) to correlate with clinical outcomes.

Ethical approval was obtained from National Research Ethics Service, London and informed consent in accordance with the Declaration of Helsinki. Out of 286 patients (2009–2014) tested at our centre using FISH on CD138+ bone marrow cells (Miltenyi microbead isolation), del(17p) was detected in 10 out of 98 (10.2%) ND and 42 out of 188 (22.3%) relapsed patients. An additional 6 patients were diagnosed with del(17p) at another centre. Material was available for all 16 ND and 35 relapse patients for mutational screening. In 23 patients, samples were available at more than one time point (range 2–4). FISH analysis was performed using standard probes.^11^ Genomic DNA was amplified by polymerase chain reaction and screened for *TP53* mutations in exons 4–11 using denaturing high-performance liquid chromatography (DHPLC; Transgenomic, Glasgow, UK). Mutations were verified by Sanger sequencing. Extended mutation testing was performed on *TP53-*mutated samples using targeted next generation sequencing (NGS; Supplementary Materials). Clinical details including treatment and disease response (2014 IMWG criteria) were collated. Survival was estimated using Kaplan–Meier methods, and differences were assessed by log-rank test, with *P*-values <0.05 considered statistically significant.

Eighteen different *TP53* mutations were identified in 18 del(17p) patients (35%) by DHPLC (Table 1). Consistent with other studies,^10^ most mutations occurred in exons 5, 6 and 8 encoding an integral part of the p53 DNA binding domain. Seventeen (94%) were single-nucleotide variants (SNVs), a similar spectrum to that reported previously,^10^ although others have found deletions or insertions.^2^ Seventeen SNVs were documented on the International Agency for Research on Cancer *TP53* database; 14 (82%) were missense mutations, two nonsense mutations and one altered a splice site. Most were predicted to produce a non-functional protein with <20% transcriptional activity. The remaining mutation was a novel in-frame indel. In 83% of samples, the variant allele frequency of the *TP53* mutation was >50%, consistent with the known del(17p) in a significant proportion of cells. In three patients, additional low-level *TP53* mutations were detected by NGS.

Twenty-three patients were tested more than once, including 8 patients with *TP53* mutations, allowing us to explore the chronology of these genetic events. *TP53* mutations were identified at multiple time points in 5 patients (Table 1). In case 1, the mutant level increased with disease progression, indicating selection of the *TP53*-mutated clone. Case 11 showed clonal evolution with the appearance of a *FAM46C* mutation at relapse. Three patients (cases 3, 4 and 9) had consistently high mutant levels at both time points. The subclonal nonsense mutation detected in case 3 was only present in the first mutated sample. Thirteen of the 18 *TP53*-mutated patients (72%) were mutant-positive in the first sample analyzed (Figure 1a). In five this was at diagnosis, indicating that 31% (5 of 16) of patients in this cohort presented with both del(17p) and a *TP53* mutation (cases 11, 15–18). However, this frequency may be an underestimate as diagnostic samples were not available from two patients who presented with del(17p), both of whom were *TP53*-mutated in a subsequent relapse sample (cases 5 and 10). In six patients, it was not possible to determine the chronology of their acquisition as earlier FISH results or stored samples were not available. In cases 1, 3 and 6, *TP53* mutations were only detected at relapse despite mutational testing in earlier samples. Two of these patients also only acquired the del(17p) at relapse. Of note, in both the remaining two patients (cases 2 and 7), del(17p) acquisition clearly preceded the *TP53* mutation. Case 2 acquired del(17p) at first relapse 34 months post-diagnosis but only acquired a *TP53* mutation at their fourth relapse 73 months post-diagnosis. Case 7 presented with del(17p), was mutant-negative at relapse 17 months later, but acquired a *TP53* mutation at second relapse 26 months post-diagnosis. The chronology of FISH and mutational testing for the 33 del(17p) patients without *TP53* mutations is shown in Supplementary Figure S1.

There was no difference in age at diagnosis or disease isotype between *TP53*-mutated and non-mutated patients (Supplementary Table S1). Ten *TP53-*mutated patients (56%) also had at least one other high-risk cytogenetic feature detected at some point in their disease (t(4;14), t(14;16) or 1q gain; Table 1), but this frequency was not significantly different from the non-*TP53-*mutated patients (56 vs 49%, *P*=0.6). Ten *TP53-*mutated patients (56%) also had additional mutations in previously reported myeloma driver genes^9, 12^ [DIS3(5), FAM46C(3), NRAS(3), KRAS(2), TRAF3(2), FGFR3(1), PRDM1(1), ATM(1), BRAF(1) and PIK3CA(1)] and most of these patients (7, 39%) had two or more additional mutations (Table 1); in our series, the incidence of *DIS3* mutations (28%) appears higher.

Median overall survival (OS) from diagnosis was significantly shorter in *TP53*-mutated than in non-mutated patients (19 vs 74 months, *P*=0.02; Supplementary Figure S2), as was median OS from the time at which del(17p) was first detected (8 vs 29 months, *P*<0.01; Figure 1b). Median progression-free survival (PFS) from first detection of del(17p) was similar between the two groups (7 vs 12 months, *P*=0.51; Supplementary Figure S3A), but the acquisition of a *TP53* mutation was associated with extremely poor survival. Median PFS of patients from the time a mutation was first detected was 5 months (Supplementary Figure S3B), and median OS was 7 months (Figure 1c). In contrast, patients who remained without detectable *TP53* mutation had a median PFS of 11.5 months, with median OS 17 months, calculated from the date of the last sample tested. There was thus a striking difference in survival between patients with and without *TP53* mutations. These results confirm that, although homozygous deletion of *TP53* in MM is uncommon,^3^ inactivating *TP53* mutations that are likely to produce non-functional proteins are frequent in del(17p) MM. *TP53* haploinsufficiency in MM causes downstream p53 pathway deregulation, altering the delivery of an apoptotic response,^6^ but the biological consequence of the additional functional loss conferred by mutation of the remaining allele remains unclear. In this cohort, we observed that *TP53* mutation usually occurred after, or simultaneously with, allelic loss of 17p13, and conferred a significantly poorer prognosis for those patients with both abnormalities. Bi-allelic inactivation of tumor suppressor genes, including *TP53*, has been reported to drive relapse in MM. A recent longitudinal study similarly identified that bi-allelic events leading to complete *TP53* inactivation resulted in the poorest prognosis at relapse.^13^ Patients with the lowest levels of *TP53* mRNA have also been reported to have the worst clinical outcome.^3, 14^ There is also evidence of other mechanisms leading to p53 inactivation in MM, including promoter methylation^6^ and aberrant expression and function of p53-regulating miRNAs,^15^ thus investigation of *TP53* mRNA and p53 protein levels may be warranted. Collectively our results confirm that, in the context of del(17p), acquisition of a *TP53* mutation on the other allele confers significantly worse clinical outcomes, and suggest that allelic loss of 17p13 likely precedes such mutations. In the era of genomic medicine, there is a case for routinely screening for *TP53* mutations in del(17p) MM to aid management decisions and direct these high-risk patients towards alternative treatment strategies aimed at counteracting the loss of tumor suppressor activity. Finally, the possibility of a different target gene for del(17p) in patients who do not have mutations of *TP53* remains to be explored.

## Figures and Tables

**Figure 1:**
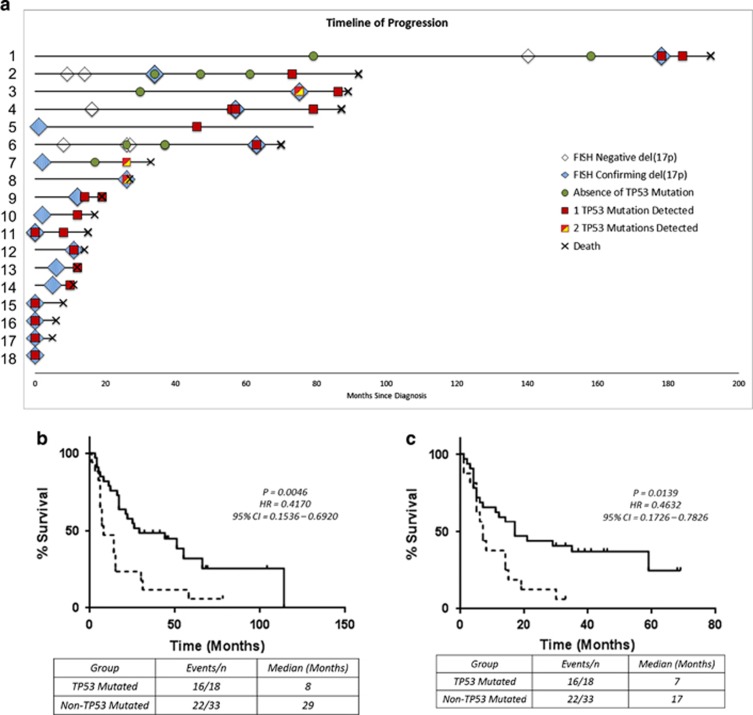
Clinical impact of *TP53* mutations. (**a**) Swimmer plot displaying the time of del(17p) FISH testing/detection and *TP53* mutation screening for each *TP53*-mutated patient. Kaplan–Meier curves for (**b**) overall survival (OS) from first detection of del(17p) in patients with and without *TP53* mutations, and (**c**) from first detection of the mutation in *TP53-*mutated patients compared to the last time point tested in non-mutated patients. Dotted Line, *TP53-*mutated patients; solid line, non-*TP53* patients. CI, 95% confidence intervals; HR, hazard ratios.

**Table 1:** Summary of TP53 mutations identified in the cohort

*ID*	*Disease status at time of mutation detection*	*Months since diagnosis*	*Del(17p) % by FISH*	*Mutated TP53 exon*	*TP53 mutation*	*TP53 mutation VAF (%)*^a^	*On IARC TP53*	*Mutational effect*^b^	*Additional mutations*^c^	*Additional FISH abnormalities*
1^d^	Relapse	178	100	7	p.C242S	52	Yes	Non-functional missense	DIS3 (14%), TRAF3 (1%)	t(4;14)
	Relapse	184	74	7	p.C242S	90	Yes	Non-functional missense	DIS3 (4%), TRAF3 (13%)	t(4;14), 13q-
2	Relapse	73	78	6	p.P190L	12	Yes	Partially functional missense	None	None
3^d^	Relapse	75	53	Int/Ex 9 10	c.993+2T>C p.R342X^e^	90 12	Yes Yes	Disrupt splicing Nonsense	DIS3 (88%), FGFR3 (36%)	t(4;14), 1q+, 13q-
	Relapse	86	87	Int/Ex 9	c.993+2T>C	97	Yes	Disrupt splicing	DIS3 (99%), FGFR3 (45%)	t(4;14), 1q+,1p-
4^d^	Relapse	56	99	6	p.I195T	97	Yes	Non-functional missense	KRAS (48%), FAM46C (31%)	1q+, 13q-
	Relapse	79	99	6	p.I195T	93	Yes	Non-functional missense	KRAS (47%), FAM46C (16%)	1q+, 13q-
5	Relapse	46	79	5	p.C141Y	66	Yes	Non-functional missense	None	None
6	Relapse	63	33	6	p.H193R	32	Yes	Non-functional nonsense	NRAS (45%), DIS3 (41%)	Hyperdiploid
7	Relapse	26	86	8 6	p.R267W p.H214R^e^	64 11	Yes	Non-functional missense Non-functional missense	None	1q+
8	Relapse	26	100	5 9	p.A138_T140delinsTSGDRPA p.Q317X^e^	78 9	No Yes	In-frame indel Nonsense	ATM (27%), DIS3 (34%), 2x NRAS (9%) (12%)	1q+
9^d^	Relapse	14	80	7	p.Y234C	99	Yes	Non-functional missense	KRAS (43%)	1p-, 13q-
	Relapse	19	80	7	p.Y234C	80	Yes	Non-functional missense	KRAS (38%)	1p-, 13q-
10	Relapse	12	100	8	p.E268A	97	Yes	Non-functional missense	None	None
11^d^	NDMM	0	100	10	p.L348S	67	Yes	Non-functional missense	PRDM1 (65%), DIS3 (11%)	13q-, t(4;14), 1p-,Tetraploid
	Relapse	8	86	10	p.L348S	39	Yes	Non-functional missense	PRDM1 (38%), DIS3 (24%), FAM46C (32%)	13q-, t(4;14), 1p-, Tetraploid
12	Relapse	11	91	5	p.A161T	97	Yes	Partially functional missense	None	t(14;16), 1q+
13	Relapse	12	100	8	p.G279E	68	Yes	Non-functional missense	BRAF (30%)	1q+
14	Relapse	10	100	8	p.P278S	73	Yes	Non-functional missense	FAM46C (23%)	None
15	NDMM	0	84	6	p.Q192X	88	Yes	Missense	NRAS (36%), PIK3CA (41%), TRAF3 (45%)	t(14;16), 13q-
16	NDMM	0	96	4	p.E62X	91	Yes	Nonsense	None	None
17	NDMM	0	92	5	p.V172D	98	Yes	Non-functional missense	None	t(11;14)
18	NDMM	0	77	4	p.F109V	47	Yes	Non-functional missense	None	t(14;16), 1q+

Abbreviations: FISH, fluorescence *in situ* hybridization; Int/Ex, intron/exon boundary; NDMM, newly diagnosed multiple myeloma; NGS, next generation sequencing; VAF, variant allele frequency.

a VAF as determined by targeted NGS.

b Taken from the International Agency for Research on Cancer (IARC) TP53 database; proteins with <20% transcriptional activity were predicted to be non-functional and with 20–75% transcriptional activity partially functional.

c Details are provided in Supplementary Table S3.

d Patients with longitudinal samples.

e Mutation only detected by targeted NGS.
