# Supplementary material for: Prevalence and timing of TP53 mutations in del(17p) myeloma and effect on survival
Source: Blood Cancer J. 2017 Sep 15;7(9):e610–. doi: 10.1038/bcj.2017.76 (PMC5637106; doi:10.1038/bcj.2017.76)
Supplement: Supplementary Materials [file bcj201776x1.docx]

**Supplementary Materials**

**Supplementary Methods: Extended Mutation Testing**

*TP53* mutant multiple myeloma samples were screened for mutations using a diagnostic amplicon panel comprising 145 amplicons covering mutation hotspots or entire coding exons of 28 genes frequently mutated in lymphoid malignancy (Supplementary Table S2). Briefly, amplicon libraries were generated from 50ng genomic DNA using the Fluidigm Access Array System (Fluidigm, CA, USA), including the addition of a sample-specific index for multiplex sequencing. Sequencing was performed on a MiSeq (Illumina, CA, USA) using 150bp paired-end reads followed by sample demultiplexing and generation of Fastq files using CASAVA software (Illumina). An in-house developed bioinformatics pipeline performed alignment and variant calling, in which primer sequences were used to assign reads to their respective amplicon followed by alignment to the reference genome (GRCh37 assembly) using a modified Smith-Waterman algorithm and identification of sequence variations using VarScan2.

All samples were also screened for mutations using a custom SureSelect hybridisation-based capture (Agilent, CA, USA) targeting all coding exons of an additional 14 genes implicated in multiple myeloma (Supplementary Table S2). DNA (200ng) was sheared using focused acoustic sonication (Covaris, MA, USA) and fragment libraries prepared using the KAPA hyper prep kit according to standard protocols (KAPA Biosystems, MA, USA). Hybridisation capture was performed according to recommended protocols (Agilent). Indexed libraries were sequenced on an Illumina NextSeq (paired-end 75bp reads) and sequence data processed through the Illumina CASAVA software to split index reads and generate Fastq files. Data was aligned to the human genome (GRCh37 assembly) using BWA-MEM^1^, locally realigned around indels (using GATK software^2^), and duplicate reads marked using Picard (http://broadinstitute.github.io/picard/). Variants were called using Unified Genotyper and Haplotype caller^2^ and annotated with information from Ensembl Variant Effect Predictor (v78).

Variants detected by both amplicon and hybridisation-based targeted next generation sequencing were manually inspected in IGV^3^ to remove sequencing errors and filtered to remove known polymorphisms (<1% in the 1000 Genomes, ExAC or EVS databases)^4-6^. Variants predicted to alter the protein sequence were curated for pathogenicity including previous description in the literature and cancer databases (e.g. COSMIC) and *in silico* prediction. Only variants considered pathogenic are reported.

**Supplementary Table S1: Clinical Details of Whole Cohort**

|  | ***TP53* Mutated (n=18)** | **Non-*TP53* Mutated**  **(n=33)** |
| --- | --- | --- |
| **Median Age at Diagnosis** | 61.1 years (range: 43.1 – 86.3) | 57.3 years (range: 28.2 – 97.4) |
| **Sex:** Female  Male | 44% 56% | 42%  58% |
| **MM Isotype:**  IgG Kappa  IgG Lambda  IgA Kappa/Lambda  Light Chain  Other | 44%  11%  22%  22% 0% | 27%  24%  12%  30%  6% |
| **Median % del(17p)*** | 91.5%  (range: 33 – 100%) | 70%  (range: 5 – 100%) |

*Del(17p) % was available in 16 of 18 *TP53* mutated patients and 33 non-*TP53* mutated patients. Median % del(17p) was calculated from the first available FISH result positive for del(17p) in non-*TP53* patients with serial samples and from the first mutated sample for *TP53-*mutated patients.

**Supplementary Table S2: Genes Analysed by Next Generation Sequencing**

| **Amplicon panel** | **Hybridisation-based panel** |
| --- | --- |
| *AKT1* (exon 3), *BIRC3* (exon 6-9), *BRAF* (exon 11, 15), *BTK* (exon 15), *CARD11* (exon 4-9), *CXCR4* (exon 2), *DNMT3A* (exon 23), *EZH2* (exon 16, 18), *FOXO1* (exon 1), *FYN* (exon 7), *IDH1* (exon 4), *IDH2* (exon 4), *JAK3* (exon 13, 15), *KRAS* (exon 2-4), *MYD88* (exon 5), *NOTCH1* (exon 26-28, 34), *NRAS* (exon 2-4), *PHF6* (exon 7-10), *PIK3CA* (exon 10, 21), *PLCG1* (exon 11), *PLCG2* (exon 19, 20, 24), *RHOA* (exon 2), *RUNX1* (exon 4-9), *SF3B1* (exon 14-16), *STAT3* (exon 21), *STAT5B* (exon 16), *STAT6* (exon 10, 13, 16), *TP53* (exon 2-11) | *ATM*, *ATR*, *CCND1*, *CYLD*, *DIS3*, *FAM46C*, *FGFR3*, *IRF4*, *NCKAP5*, *PRDM1*, *PRKD2*, *RB1*, *TRAF3*, *ZFHX4* |

**Supplementary Table S3: Mutations Identified by Extended Sequencing**

| **ID** | **Gene** | **HGVSg** | **HGVSc** | **HGVSp** |
| --- | --- | --- | --- | --- |
| 1 | DIS3 | chr13:g.73342974C>T | NM_014953.3:c.1832G>A | NP_055768.3:p.Arg611His |
|  | TRAF3 | chr14:g.103342779C>T | NM_145725.2:c.487C>T | NP_663777.1:p.Arg163* |
| 3 | FGFR3 | chr4:g.1806088_1806089delinsAT | NM_000142.4:c.1107_1108delinsAT | NP_000133.1:p.Gly370Cys |
|  | DIS3 | chr13:g.73350139C>A | NM_014953.3:c.746G>T | NP_055768.3:p.Gly249Val |
| 4 | FAM46C | chr1:g.118166053T>C | NM_017709.3:c.563T>C | NP_060179.2:p.Leu188Pro |
|  | KRAS | chr12:g.25380275T>G | NM_033360.2:c.183A>C | NP_203524.1:p.Gln61His |
| 6 | DIS3 | chr13:g.73345240G>A | NM_014953.3:c.1649C>T | NP_055768.3:p.Ser550Phe |
|  | NRAS | chr1:g.115258748C>T | NM_002524.4:c.34G>A | NP_002515.1:p.Gly12Ser |
| 8 | ATM | chr11:g.108213956C>G | NM_000051.3:c.8276C>G | NP_000042.3:p.Pro2759Arg |
|  | DIS3 | chr13:g.73350115T>A | NM_014953.3:c.770A>T | NP_055768.3:p.Asn257Ile |
|  | NRAS | chr1:g.115258748C>G | NM_002524.4:c.34G>C | NP_002515.1:p.Gly12Arg |
|  | NRAS | chr1:g.115258747C>A | NM_002524.4:c.35G>T | NP_002515.1:p.Gly12Val |
| 9 | KRAS | chr12:g.25398285C>G | NM_033360.2:c.34G>C | NP_203524.1:p.Gly12Arg |
| 11 | FAM46C | chr1:g.118166034G>T | NM_017709.3:c.544G>T | NP_060179.2:p.Asp182Tyr |
|  | PRDM1 | chr6:g.106554982_106554983del | NM_001198.3:c.2099_2100del | NP_001189.2:p.Cys700* |
|  | DIS3 | chr13:g.73336166C>T | NM_014953.3:c.2237G>A | NP_055768.3:p.Arg746His |
| 13 | BRAF | chr7:g.140453136A>T | NM_004333.4:c.1799T>A | NP_004324.2:p.Val600Glu |
| 14 | FAM46C | chr1:g.118165764G>T | NM_017709.3:c.274G>T | NP_060179.2:p.Asp92Tyr |
| 15 | NRAS | chr1:g.115258747C>G | NM_002524.4:c.35G>C | NP_002515.1:p.Gly12Ala |
|  | PIK3CA | chr3:g.178936082G>A | NM_006218.2:c.1624G>A | NP_006209.2:p.Glu542Lys |
|  | TRAF3 | chr14:g.103363706C>T | NM_145725.2:c.928C>T | NP_663777.1:p.Arg310* |

**Supplementary Figure S1: Clinical timeline of del(17p) MM patients.** Swimmer plot displaying the time of first del(17p) detection by FISH and *TP53* mutation screening for each non *TP53*-mutated patient.


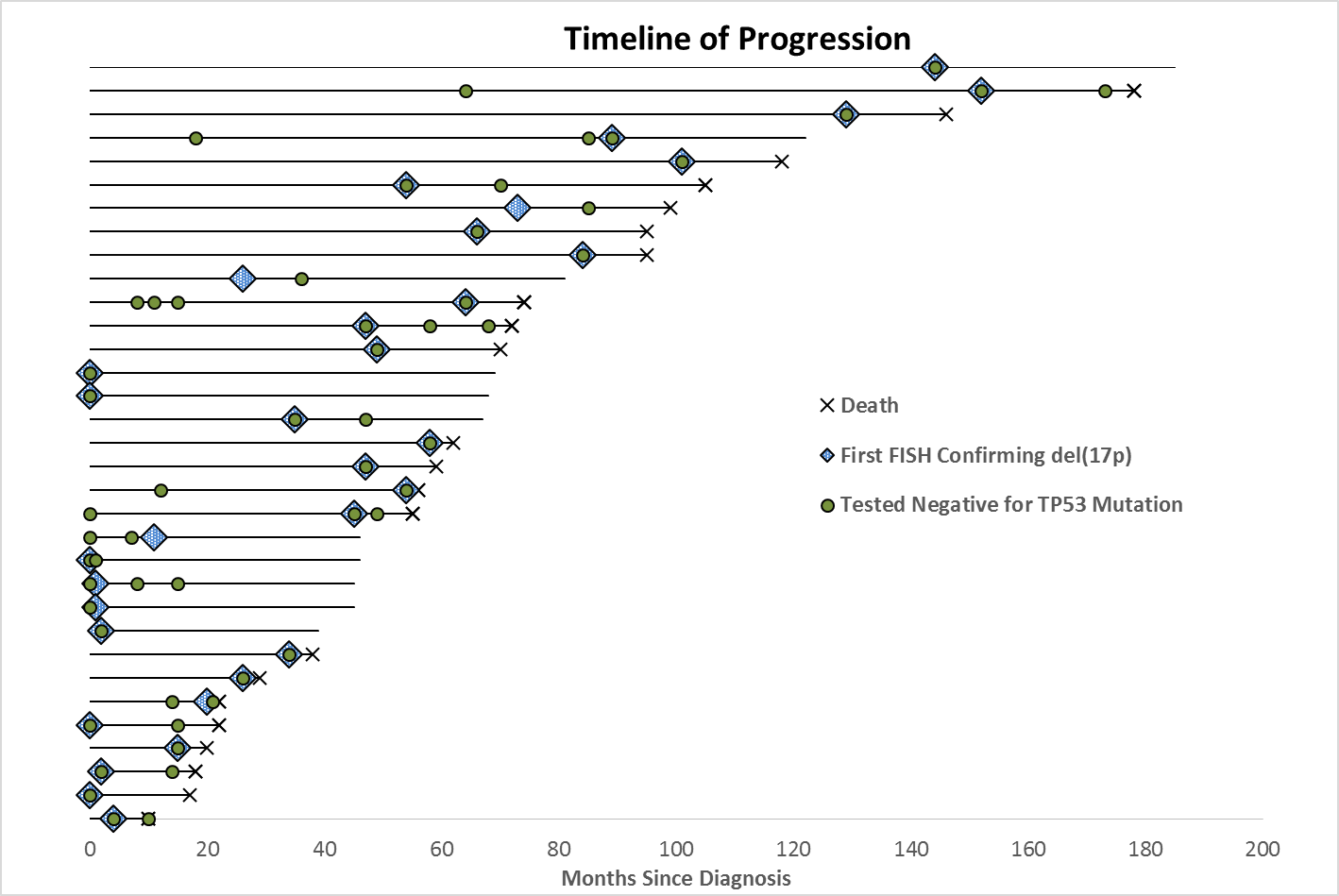
**Supplementary Figure S2: OS from diagnosis comparing del(17p) patients with and without *TP53* mutations**


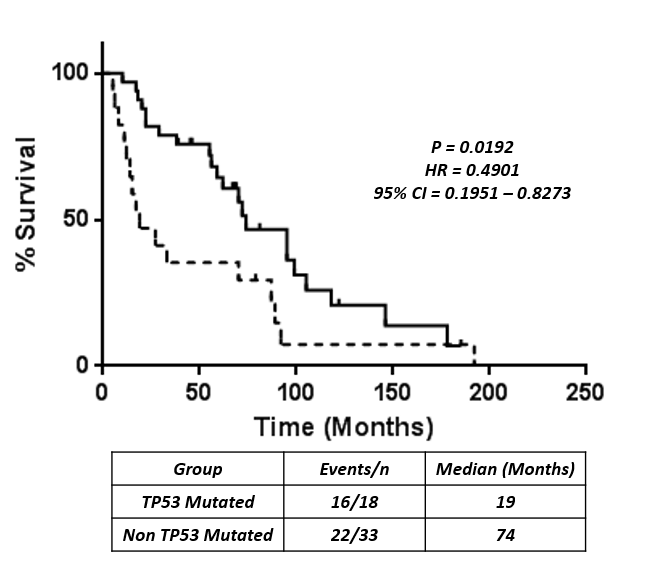


Kaplan-Meier curve showing overall survival (OS) from diagnosis. Dotted Line = *TP53-*mutated patients; Solid Line = non-*TP53* mutated patients. HR, Hazard ratios; CI, 95% confidence intervals.

**Supplementary Figure S3: PFS comparing del(17p) patients with and without *TP53* mutations**
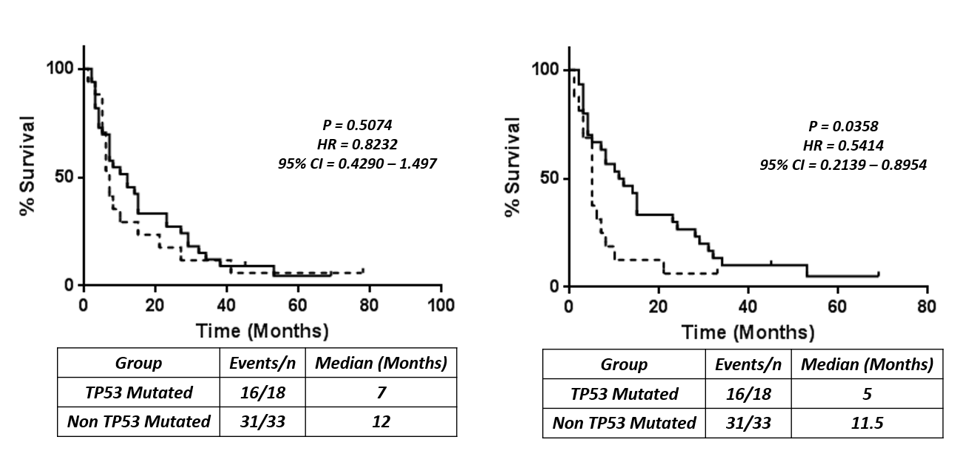


Kaplan-Meier curves showing progression-free survival (PFS) (A) from first detection of del(17p), (B) from first detection of the *TP53* mutation compared to last tested time point in non-mutated patients. Dotted Line = *TP53*-mutated patients; Solid Line = non-*TP53* mutated patients. HR, Hazard ratios; CI, 95% confidence intervals.

**Supplementary References**

1. Li H, Durbin R. Fast and accurate short read alignment with Burrows-Wheeler transform. *Bioinformatics* 2009; **25**: 1754–1760.
2. McKenna A, Hanna M, Banks E, Sivachenko A, Cibulskis K, Kernytsky A *et al.* The Genome Analysis Toolkit: a MapReduce framework for analyzing next-generation DNA sequencing data. *Genome Res* 2010; **20**: 1297–1303.
3. Thorvaldsdóttir H, Robinson JT, Mesirov JP. Integrative Genomics Viewer (IGV): high-performance genomics data visualization and exploration. *Brief Bioinform* 2013; **14**: 178–192.
4. 1000 Genomes Project Consortium, Auton A, Brooks LD, Durbin RM, Garrison EP, Kang HM *et al.* A global reference for human genetic variation. *Nature* 2015; **526**: 68–74.
5. Lek M, Karczewski KJ, Minikel EV, Samocha KE, Banks E, Fennell T *et al.* Analysis of protein-coding genetic variation in 60,706 humans. *Nature* 2016; **536**: 285–291.
6. Exome Variant Server. NHLBI GO Exome Sequencing Project (ESP). http://evs.gs.washington.edu/EVS/.
